# Associations of leptin and leptin receptor genetic variants with coronary artery disease: a meta-analysis

**DOI:** 10.1042/BSR20190466

**Published:** 2019-06-10

**Authors:** Peilin Xiao, Jianli Shi, Xiaoli Liu

**Affiliations:** Department of Cardiology, The Second Affiliated Hospital of Chongqing Medical University, Chongqing 400010, China

**Keywords:** Coronary artery disease (CAD), Gene variants, Leptin (LEP), Leptin receptor (LEPR), Meta-analysis

## Abstract

**Background:** Some pilot studies already tried to investigate potential associations of leptin (*LEP*) and LEP receptor (*LEPR*) variants with coronary artery disease (CAD). However, the results of these studies were not consistent. Thus, we performed the present meta-analysis to explore associations between *LEP/LEPR* variants and CAD in a larger pooled population.

**Methods:** Systematic literature research of PubMed, Web of Science, Embase and CNKI was performed to identify eligible case–control studies on associations between *LEP/LEPR* variants and CAD. The initial search was conducted in September 2018 and the latest update was performed in December 2018. Q test and *I^2^* statistic were employed to assess between-study heterogeneities. If probability value(*P*-value) of Q test was less than 0.1 or *I^2^* was greater than 50%, random-effect models (REMs) would be used to pool the data. Otherwise, fixed-effect models (FEMs) would be applied for synthetic analyses.

**Results:** A total of ten studies published between 2006 and 2018 were eligible for analyses (1989 cases and 2601 controls). Pooled analyses suggested that *LEP* rs7799039 variant was significantly associated with CAD under over-dominant model (*P*=0.0007, odds ratio (OR) = 1.36, 95% confidence interval (CI): 1.14–1.63, *I^2^* = 41%, FEM) in overall population, and this significant finding was further confirmed in East Asians in subsequent subgroup analyses. However, no positive findings were observed for *LEPR* rs1137100 and rs1137101 variants in overall and subgroup analyses.

**Conclusions:** Our meta-analysis suggested that *LEP* rs7799039 variant might affect individual susceptibility to CAD.

## Introduction

Coronary artery disease (CAD) is the primary cause of mortality and morbidity worldwide [1,2]. However, despite its high prevalence, the exact pathogenesis of CAD is still not fully understood. Recently, growing evidence supports that genetic factors are crucial for its development. To begin with, family aggregation of CAD was extremely common, and past twin studies proved that hereditary factors accounted for approximately 50% of CAD occurrence [3,4]. Moreover, previous genetic association studies already identified numerous genetic variants that were associated with an increased susceptibility to CAD, and screening of common causal variants also proved to be a cost-efficient way to predict the individual risk of developing CAD [5,6]. In summary, these findings jointly supported that genetic predisposition factors played vital roles in the pathogenesis of CAD.

Leptin (LEP), a hormone that is primarily secreted by adipocytes, plays a pivotal role in regulating food intake, energy homeostasis and lipid metabolism by binding with LEP receptor (LEPR) [7,8]. Previous studies showed that serum levels of LEP were significantly elevated in patients with obesity, diabetes, insulin resistance, hypertension and atherosclerosis, suggesting that LEP/LEPR might be involved in pathogenesis of above-mentioned diseases [9–11]. Considering the close relationship between these disorders and CAD, it is possible that functional *LEP/LEPR* variants, which may result in altered LEP levels, may also impact individual susceptibility to CAD.

Previous studies showed that functional *LEP/LEPR* variants were correlated with higher plasma levels of LEP [12,13]. As a result, some pilot studies tried to investigate potential associations between *LEP/LEPR* variants and CAD. But the results of these studies were not consistent. Thus, we performed the present meta-analysis to explore associations between *LEP/LEPR* variants and CAD in a larger pooled population.

## Materials and methods

### Literature search and inclusion criteria

In accordance with Reporting Items for Systematic Reviews and Meta-analyses (PRISMA) guideline, eligible studies were retrieved from PubMed, Web of Science, Embase and CNKI using the following searching strategy: (leptin receptor OR LEPR OR leptin OR LEP) AND (polymorphism OR variant OR variation OR mutation OR genotype OR allele) AND (coronary heart disease OR coronary artery disease OR angina pectoris OR acute coronary syndrome OR myocardial infarction) [14]. The initial search was conducted in September 2018 and the latest update was performed in December 2018. Moreover, we also checked the references of eligible articles to identify other potential relevant studies.

Included studies should meet all the following criteria: (i) case–control study about *LEP/LEPR* variants and CAD; (ii) provide genotypic frequency of investigated variants in cases and controls; (iii) full text in English or Chinese available. Studies were excluded if one of the following criteria was fulfilled: (i) not relevant to *LEP/LEPR* variants and CAD; (ii) *in vitro *studies or animal studies; (iii) case reports or case series; (iv) abstracts, reviews, comments, letters and conference presentations.

### Data extraction and quality assessment

We extracted following data from the included studies: (i) the name of the first author; (ii) publication time; (iii) country and ethnicity; (iv) sample size; and (v) genotypic distributions of *LEP/LEPR* variants in cases and controls. The probability value (*P-*value) of Hardy–Weinberg equilibrium (HWE) was also calculated. When necessary, we wrote to the corresponding authors for extra information. We used the Newcastle–Ottawa scale (NOS) to assess the quality of eligible studies [15]. This scale has a score range of 0–9, and studies with a score of more than 7 were thought to be of high quality. Data extraction and quality assessment were performed by two independent reviewers (Peilin Xiao and Jianli Shi). Any disagreement between two reviewers was solved by discussion until a consensus was reached.

### Statistical analyses

We used Review Manager Version 5.3.3 (The Cochrane Collaboration, Software Update) to conduct statistical analyses. We calculated odds ratios (ORs) and 95% confidence intervals (CIs) to estimate strength of associations in all possible genetic models, and *P*-values ≤0.05 were considered to be statistically significant. All investigated *LEP/LEPR* variants contain a major allele (M) and a minor allele (m), the dominant comparison is defined as MM versus Mm + mm, recessive comparison is defined as mm vs. MM + Mm, over-dominant comparison is defined as Mm versus MM + mm, and the allele comparison is defined as M versus m. Q test and *I^2^* statistic were employed to assess between-study heterogeneities. If *P*-value of Q test was less than 0.1 or *I^2^* was greater than 50%, random-effect models (REMs) would be used to pool the data. Otherwise, fixed-effect models (FEMs) would be used to pool the data. Subgroup analyses by ethnicity of participants and type of disease were performed. Sensitivity analyses were performed to evaluate the statistical robustness of the findings, and publication biases were evaluated with funnel plots.

## Results

### Characteristics of included studies

**Figure 1:**
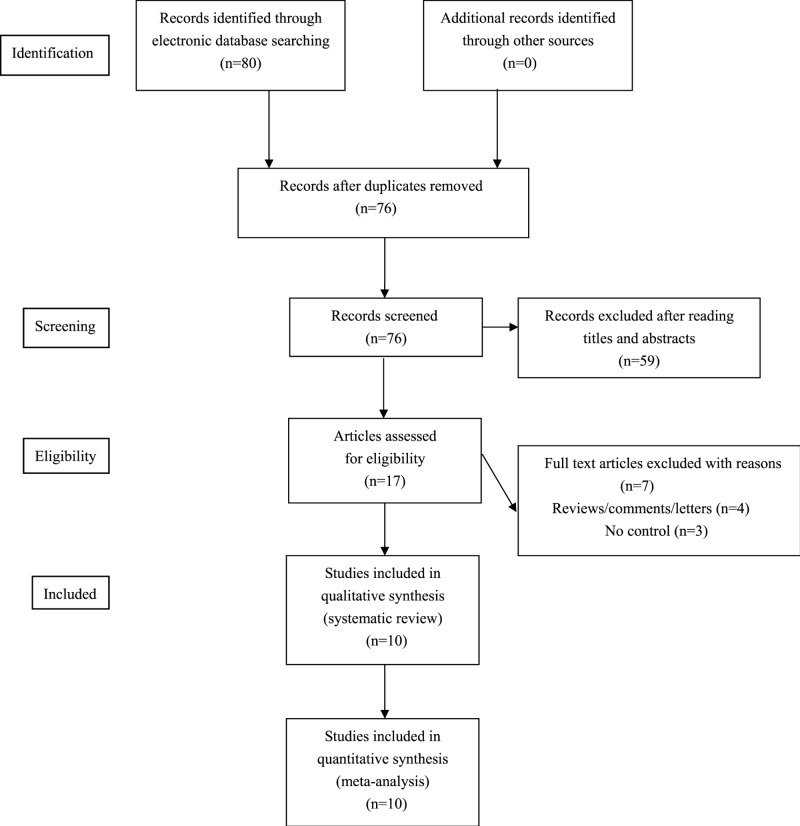
Flow chart of study selection for the present study

We found 80 potential relevant articles. Among these articles, a total of ten studies published between 2006 and 2018 were eligible for pooled analyses [16–25] (see Figure 1). The NOS score of eligible articles ranged from 7 to 8, which indicated that all included studies were of high quality. Baseline characteristics of included studies were shown in Table 1.

**Table 1:** The characteristics of included studies

First author, year	Country	Ethnicity	Type of disease	Sample size	Genotype distribution		*P*-value for HWE	NOS score
					Cases	Controls		
***LEPR* rs1137100**					AA/AG/GG			
Aijälä, 2014 [16]	Finland	Caucasian	CAD	151/877	66/75/10	361/398/118	0.617	7
An, 2016 [17]	China	East Asian	CAD	421/550	295/110/16	371/157/22	0.299	7
Roszkowska-Gancarz, 2014 [20]	Russia	Caucasian	MI	226/414	118/86/22	218/171/25	0.259	7
***LEPR* rs1137101**					GG/GA/AA			
Aijälä, 2014 [16]	Finland	Caucasian	CAD	152/882	48/80/24	312/427/143	0.878	7
An, 2016 [17]	China	East Asian	CAD	421/550	306/103/12	362/162/26	0.158	7
Khaki-Khatibi, 2018 [18]	Iran	South Asian	MI	80/80	18/37/25	30/36/14	0.576	8
Nowzari, 2018 [19]	Iran	South Asian	CAD	156/130	70/61/25	63/34/33	<0.001	7
Roszkowska-Gancarz, 2014 [20]	Russia	Caucasian	MI	226/414	59/108/59	116/206/92	0.976	7
Wu, 2013 [22]	China	East Asian	CAD	200/100	42/90/68	44/38/18	0.064	8
***LEP* rs7799039**					GG/GA/AA			
Nowzari, 2018 [19]	Iran	South Asian	CAD	156/130	61/73/22	58/51/21	0.095	7
Roszkowska-Gancarz, 2014 [20]	Russia	Caucasian	MI	226/414	67/120/39	139/203/72	0.885	7
Wang, 2013 [21]	China	East Asian	CAD	200/100	65/100/35	37/46/17	0.677	8
Zhai, 2013 [23]	China	East Asian	CAD	200/100	102/86/12	45/38/17	0.080	8
Zhao, 2006 [24]	China	East Asian	CAD	156/150	80/68/8	68/56/26	0.020	8
Zheng, 2014 [25]	China	East Asian	CAD	198/95	68/97/33	65/24/6	0.084	7

Abbreviation: MI, myocardial infarction.

### Overall and subgroup analyses

Three studies about *LEPR* rs1137100 A/G variant (798 cases and 1841 controls), six studies about *LEPR* rs1137101 G/A variant (1235 cases and 2156 controls) and six studies about *LEP* rs7799039 G/A variant (1136 cases and 989 controls) were eligible for synthetic analyses. Pooled analyses showed that *LEP* rs7799039 G/A variant was significantly associated with CAD under over-dominant model (GA vs. GG + AA, *P*=0.0007, OR = 1.36, 95%CI: 1.14–1.63, *I^2^* = 41%, FEM) in overall population, and this significant finding was further confirmed in East Asians in subsequent subgroup analyses (GA vs. GG + AA, *P*=0.04, OR = 1.50, 95%CI: 1.02–2.20, *I^2^* = 59%, REM). However, no any positive findings were observed for *LEPR* rs1137100 and rs1137101 variants in overall and subgroup analyses (see Table 2 and Supplementary Figures S1–S3).

**Table 2:** Results of overall and subgroup analyses

Polymorphisms	Population	Sample size	Dominant comparison			Recessive comparison			Over-dominant comparison			Allele comparison		
		Cases/ Controls	*P* value	OR (95%CI)	*I^2^*	*P* value	OR (95%CI)	*I^2^*	*P* value	OR (95%CI)	*I^2^*	*P* value	OR (95%CI)	*I^2^*
***LEPR* rs1137100**	Overall	798/1841	0.80	0.91 (0.43–1.92)	76%	0.42	1.08 (0.90–1.29)	0%	0.64	0.96 (0.80–1.15)	4%	0.35	0.93 (0.81–1.08)	31%
	Caucasian	377/1291	0.85	0.88 (0.24–3.20)	88%	0.75	1.04 (0.82–1.32)	0%	0.93	1.01 (0.80–1.28)	37%	0.74	0.95 (0.70–1.29)	65%
***LEPR* rs1137101**	Overall	1235/2156	0.17	0.76 (0.52–1.13)	81%	0.62	1.12 (0.72–1.72)	73%	0.49	1.09 (0.86–1.38)	53%	0.31	0.85 (0.63–1.16)	85%
	East Asian	621/650	0.61	0.70 (0.18–2.76)	95%	0.80	1.19 (0.31–4.61)	89%	0.94	0.98 (0.58–1.66)	71%	0.67	0.79 (0.27–2.32)	96%
	South Asian	236/210	0.10	0.72 (0.49–1.06)	46%	0.92	1.07 (0.29–3.99)	87%	0.06	1.46 (0.99–2.16)	44%	0.62	0.83 (0.39–1.75)	86%
	Caucasian	378/1296	0.31	0.87 (0.67–1.13)	0%	0.44	1.12 (0.84–1.50)	0%	0.76	1.04 (0.82–1.31)	5%	0.27	0.91 (0.77–1.08)	0%
	MI	306/494	0.27	0.71 (0.39–1.30)	60%	0.05	1.39 (0.99–1.94)	40%	0.73	0.95 (0.71–1.27)	0%	0.18	0.73 (0.47–1.15)	70%
***LEP* rs7799039**	Overall	1136/989	0.27	0.78 (0.50–1.21)	82%	0.38	0.77 (0.43–1.38)	78%	**0.0007**	**1.36 (1.1**4**–1.63)**	41%	0.67	0.92 (0.64–1.34)	87%
	East Asian	754/445	0.46	0.76 (0.36–1.59)	89%	0.50	0.70 (0.25–1.96)	86%	**0.04**	**1.50 (1.02–2.20)**	59%	0.79	0.92 (0.48–1.75)	92%

The values in bold represent statistically significant differences between cases and controls. Abbreviation: MI, myocardial infarction.

### Sensitivity analyses

We performed sensitivity analyses to test stabilities of pooled results by excluding one study each time. No altered results were observed in overall and subgroup comparisons, which indicated that our findings were statistically stable.

### Publication biases

We used funnel plots to assess publication biases. We did not find obvious asymmetry of funnel plots in any comparisons, which suggested that our findings were unlikely to be impacted by severe publication biases (see Supplementary Figures S4–S6).

## Discussion

As far as we know, this is the first meta-analysis on associations of *LEP/LEPR* variants with CAD, and our pooled analyses suggested that *LEP* rs7799039 G/A variant might affect individual susceptibility to CAD. There are several notable points about this meta-analysis. First, there are two possible explanations for our positive findings regarding *LEP* rs7799039 G/A variant and CAD. First, this variant may lead to alteration in gene expression or change in LEP protein structure, which may subsequently affect biological functions of LEP, result in elevated level of LEP and ultimately impact individual susceptibility to CAD. Second, it is also possible that *LEP/LEPR* variants may be linked to each other or even linked to other unidentified genes, which could also impact individual susceptibility to CAD [26]. Second, it is worth noting that the functional significances of two investigated *LEPR* variants were also well established [27,28], yet no significant associations were observed for these two variants. Since the sample sizes of pooled analyses in the current meta-analysis were still relatively small, it is possible that our study was still not statistically adequate to detect the actual associations between *LEP/LEPR* variants and CAD in every genetic comparison. Therefore, further studies with larger sample sizes still need to test the associations between *LEPR* variants and CAD. Third, the pathogenesis of CAD is extremely complex, and therefore the probability that a specific genetic variant could significantly contribute to its development is low, and we strongly recommend further studies to perform haplotype analyses and explore potential gene–gene interactions. Fourth, to more precisely measure the effects of certain endogenous/exogenous factors on disease occurrence and development, molecular pathologic epidemiology (MPE) analyses should be adopted. However, since included studies only focused on the effects of *LEP/LEPR* variants on individual susceptibility to CAD, such analyses were infeasible in the current meta-analysis. But to better elucidate the underlying pathogenesis mechanisms of CAD, future studies should try to investigate the interaction of *LEP/LEPR* variants (as endogenous factors) with potential pathogenic environmental factors (as exogenous factors) as an MPE approach [29]. Fifth, between-study heterogeneities remained significant in several subgroup comparisons, which indicated that differences in genotypic distributions of investigated variants among eligible studies could not be fully explained by differences in type of disease or ethnicity of study subjects, and other baseline characteristics of participants may also contribute to heterogeneities between studies. Sixth, the present meta-analysis aimed to investigate associations between all *LEP/LEPR* variants and CAD. Nevertheless, only three variants were analyzed because no other variants were studied by at least two eligible studies.

Like all meta-analysis, the present study certainly has some limitations. First, due to lack of raw data, adjusted analyses were inapplicable, and we have to admit that failure to perform further adjusted analyses for potential confounding factors might impact the reliability of our findings [30]. Second, associations between *LEP/LEPR* variants and CAD might also be modified by gene–environmental interactions. However, we could not perform relevant analyses accordingly since most of studies did not investigate these associations [31,32]. Considering that certain environmental factors like smoking, air pollution, lack of physical exercise and high caloric diets were already verified to be potential risk factors of CAD, potential gene–environmental interactions should also be analyzed by future genetic association studies [33]. Third, gray literatures like abstracts and other research materials that were not formally published in academic journals were not considered to be eligible for analyses in this meta-analysis since it was hard to determine their quality. However, since gray literatures were not analyzed, although funnel plots suggested that severe publication biases were unlikely, it is still possible that our findings may be impacted by potential publication biases [34]. On account of above-mentioned limitations, our findings should be cautiously interpreted.

In conclusion, our meta-analysis suggested that *LEP* rs7799039 variant might affect individual susceptibility to CAD. However, further well-designed studies with larger sample sizes still need to confirm our findings. Moreover, future investigations are also warranted to explore potential roles of other *LEP/LEPR* variants in the development of CAD.

## Supporting information

**Supplementary Figure S1-S6:** 

## Abbreviations

CAD: coronary artery disease
CI: confidence interval
FEM: fixed-effect model
LEP: leptin
LEPR: LEP receptor
MPE: molecular pathologic epidemiology
NOS: Newcastle–Ottawa scale
OR: odds ratio
*P*-value: probability value
REM: random-effect model
