# Supplementary material for: Associations of leptin and leptin receptor genetic variants with coronary artery disease: a meta-analysis
Source: Biosci Rep. 2019 Jun 10;39(6):BSR20190466. doi: 10.1042/BSR20190466 (PMC6558721; doi:10.1042/BSR20190466)
Supplement: Supplementary file 1 [file bsr-2019-0466_suppS1.pdf]

Supplementary figure 1-Forest plots of LEPR rs1137100

Study or Subgroup	Experimental Events	Experimental Total	Control Events	Control Total	Weight	Odds Ratio M-H, Random, 95% CI
Aijälä 2014	10	151	118	877	32.6%	0.46 [0.23, 0.89]
An 2016	16	421	22	550	32.9%	0.95 [0.49, 1.83]
Roszkowska-Gancarz 2014	22	226	25	414	34.4%	1.68 [0.92, 3.05]
**Total (95% CI)**		**798**		**1841**	**100.0%**	**0.91 [0.43, 1.92]**
Total events	48		165			
Heterogeneity: Tau² = 0.33; Chi² = 8.21, df = 2 (P = 0.02); I² = 76%						
Test for overall effect: Z = 0.25 (P = 0.80)						

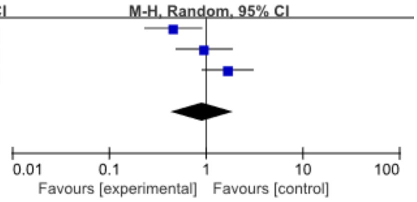

Forest plot of the ***LEPR*** **rs1137100** polymorphism and CAD under dominant comparison

(AA vs. AG + GG).

Study or Subgroup	Experimental Events	Experimental Total	Control Events	Control Total	Weight	Odds Ratio M-H, Fixed, 95% CI
Aijälä 2014	66	151	361	877	26.0%	1.11 [0.78, 1.57]
An 2016	295	421	371	550	41.9%	1.13 [0.86, 1.49]
Roszkowska-Gancarz 2014	118	226	218	414	32.1%	0.98 [0.71, 1.36]
**Total (95% CI)**		**798**		**1841**	**100.0%**	**1.08 [0.90, 1.29]**
Total events	479		950			
Heterogeneity: Chi² = 0.45, df = 2 (P = 0.80); I² = 0%						
Test for overall effect: Z = 0.81 (P = 0.42)						

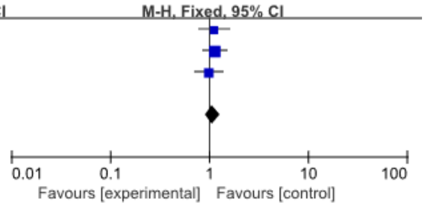

Forest plot of the ***LEPR*** **rs1137100** polymorphism and CAD under recessive comparison.

(GG vs. AA + AG)

Study or Subgroup	Experimental Events	Experimental Total	Control Events	Control Total	Weight	Odds Ratio M-H, Fixed, 95% CI
Aijälä 2014	75	151	398	877	25.1%	1.19 [0.84, 1.68]
An 2016	110	421	157	550	42.9%	0.89 [0.67, 1.18]
Roszkowska-Gancarz 2014	86	226	171	414	31.9%	0.87 [0.63, 1.22]
**Total (95% CI)**		**798**		**1841**	**100.0%**	**0.96 [0.80, 1.15]**
Total events	271		726			
Heterogeneity: Chi² = 2.08, df = 2 (P = 0.35); I² = 4%						
Test for overall effect: Z = 0.47 (P = 0.64)						

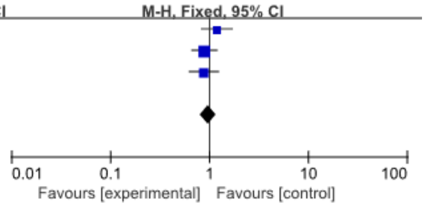

Forest plot of the ***LEPR*** **rs1137100** polymorphism and CAD under additive comparison.

(AG vs. AA + GG)

Study or Subgroup	Experimental Events	Experimental Total	Control Events	Control Total	Weight	Odds Ratio M-H, Fixed, 95% CI
Aijälä 2014	95	302	634	1754	33.3%	0.81 [0.62, 1.05]
An 2016	142	842	201	1100	37.8%	0.91 [0.72, 1.15]
Roszkowska-Gancarz 2014	130	452	221	828	29.0%	1.11 [0.86, 1.43]
**Total (95% CI)**		**1596**		**3682**	**100.0%**	**0.93 [0.81, 1.08]**
Total events	367		1056			
Heterogeneity: Chi² = 2.92, df = 2 (P = 0.23); I² = 31%						
Test for overall effect: Z = 0.93 (P = 0.35)						

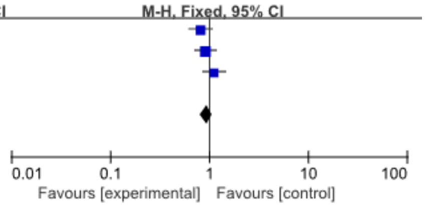

Forest plot of the ***LEPR*** **rs1137100** polymorphism and CAD under allele comparison.

(A vs. G)

Supplementary figure 2-Forest plots of LEPR rs1137101

Study or Subgroup	Experimental Events	Experimental Total	Control Events	Control Total	Weight	Odds Ratio M-H, Random, 95% CI
Aijälä 2014	48	152	312	882	18.0%	0.84 [0.58, 1.22]
An 2016	306	421	362	550	19.4%	1.38 [1.05, 1.82]
Khaki-Khatibi 2018	18	80	30	80	12.8%	0.48 [0.24, 0.97]
Nowzari 2018	70	156	63	130	16.4%	0.87 [0.54, 1.38]
Roszkowska-Gancarz 2014	59	226	116	414	18.0%	0.91 [0.63, 1.31]
Wu 2013	42	200	44	100	15.5%	0.34 [0.20, 0.57]
**Total (95% CI)**		**1235**		**2156**	**100.0%**	**0.76 [0.52, 1.13]**
Total events	543		927			

Heterogeneity: Tau² = 0.18; Chi² = 25.94, df = 5 (P < 0.0001); I² = 81%
Test for overall effect: Z = 1.36 (P = 0.17)

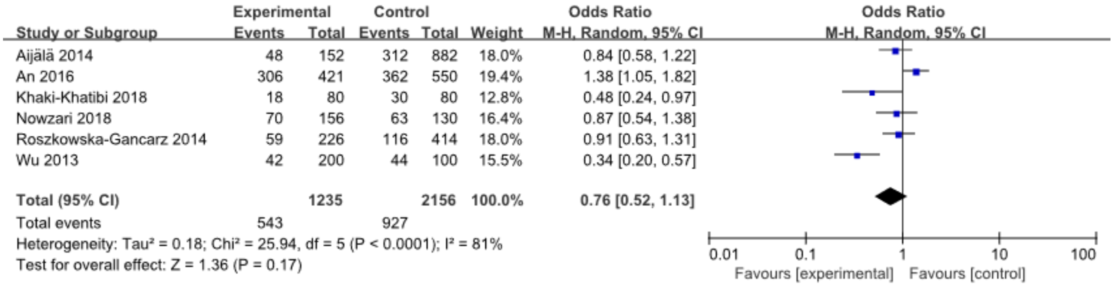

Forest plot of the ***LEPR*** **rs1137101** polymorphism and CAD under dominant comparison.

(GG vs. GA + AA)

Study or Subgroup	Experimental Events	Experimental Total	Control Events	Control Total	Weight	Odds Ratio M-H, Random, 95% CI
Aijälä 2014	24	152	143	882	18.5%	0.97 [0.60, 1.55]
An 2016	12	421	26	550	14.7%	0.59 [0.29, 1.19]
Khaki-Khatibi 2018	25	80	14	80	13.9%	2.14 [1.02, 4.52]
Nowzari 2018	25	156	33	130	16.5%	0.56 [0.31, 1.00]
Roszkowska-Gancarz 2014	59	226	92	414	20.0%	1.24 [0.85, 1.80]
Wu 2013	68	200	18	100	16.4%	2.35 [1.30, 4.23]
**Total (95% CI)**		**1235**		**2156**	**100.0%**	**1.12 [0.72, 1.72]**
Total events	213		326			

Heterogeneity: Tau² = 0.21; Chi² = 18.27, df = 5 (P = 0.003); I² = 73%
Test for overall effect: Z = 0.50 (P = 0.62)

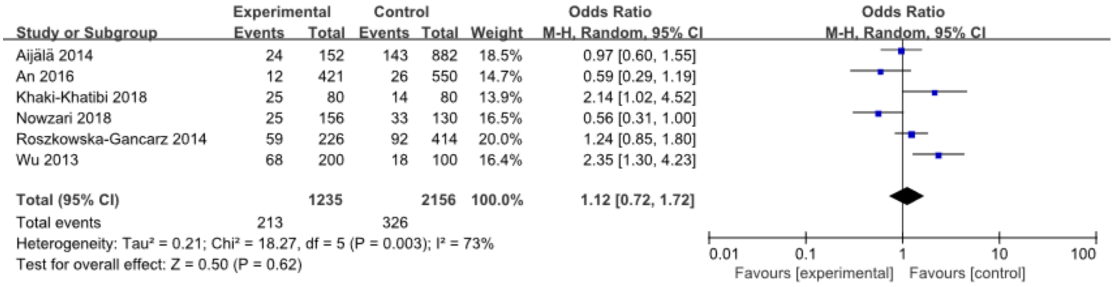

Forest plot of the ***LEPR*** **rs1137101** polymorphism and CAD under recessive comparison.

(AA vs. GG + GA)

Study or Subgroup	Experimental Events	Experimental Total	Control Events	Control Total	Weight	Odds Ratio M-H, Random, 95% CI
Aijälä 2014	80	152	427	882	19.6%	1.18 [0.84, 1.67]
An 2016	103	421	162	550	22.3%	0.78 [0.58, 1.03]
Khaki-Khatibi 2018	37	80	36	80	10.3%	1.05 [0.56, 1.96]
Nowzari 2018	61	156	34	130	13.4%	1.81 [1.09, 3.01]
Roszkowska-Gancarz 2014	108	226	206	414	20.6%	0.92 [0.67, 1.28]
Wu 2013	90	200	38	100	13.9%	1.33 [0.82, 2.18]
**Total (95% CI)**		**1235**		**2156**	**100.0%**	**1.09 [0.86, 1.38]**
Total events	479		903			

Heterogeneity: Tau² = 0.05; Chi² = 10.64, df = 5 (P = 0.06); I² = 53%
Test for overall effect: Z = 0.69 (P = 0.49)

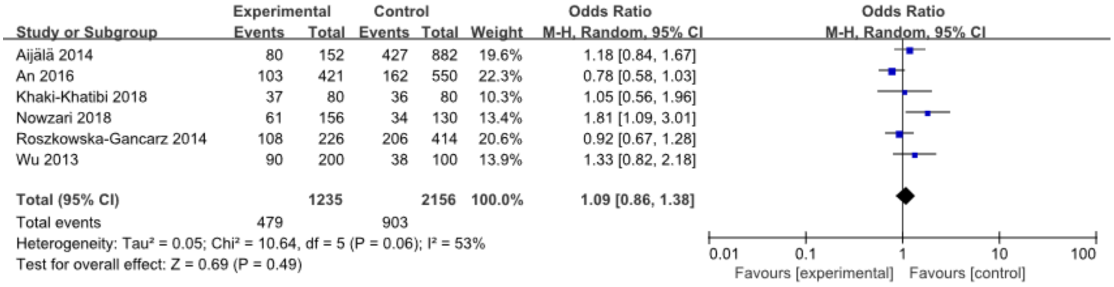

Forest plot of the ***LEPR*** **rs1137101** polymorphism and CAD under additive comparison.

(GA vs. GG + AA)

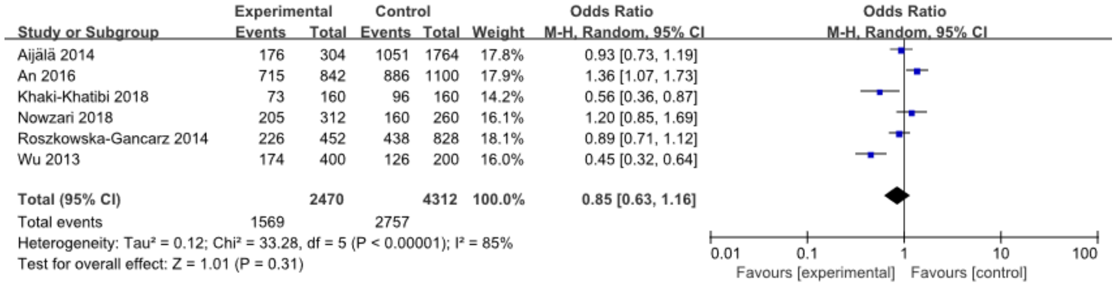

Forest plot of the ***LEPR*** **rs1137101** polymorphism and CAD under allele comparison.

(G vs. A)

Supplementary figure 3-Forest plots of LEP rs7799039

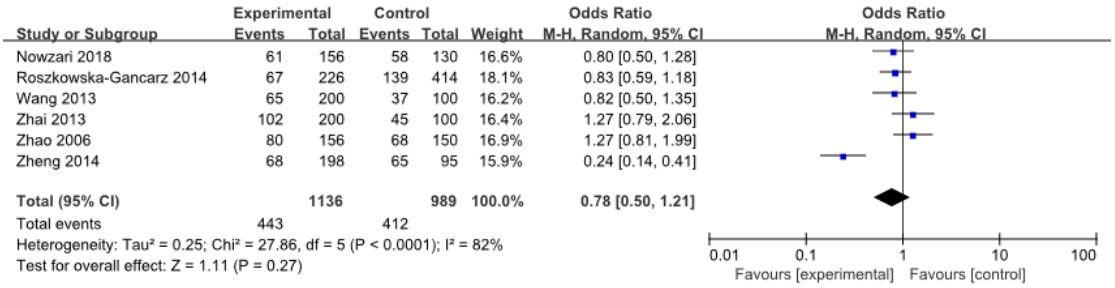

Forest plot of the ***LEP*** **rs7799039** polymorphism and CAD under dominant comparison.

(GG vs. GA + AA)

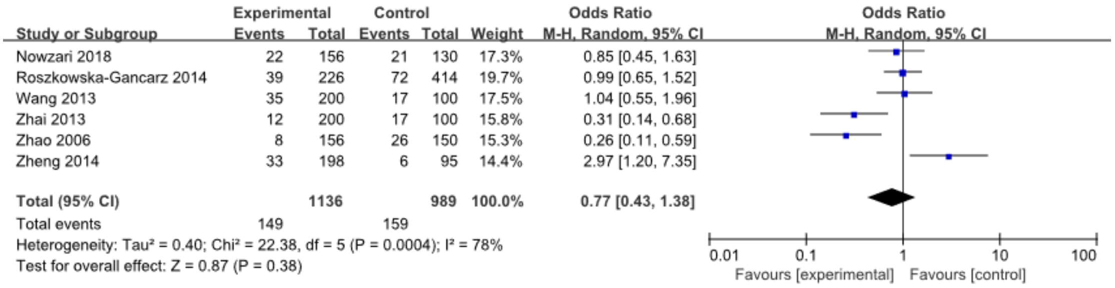

Forest plot of the ***LEP*** **rs7799039** polymorphism and CAD under recessive comparison.

(AA vs. GG + GA)

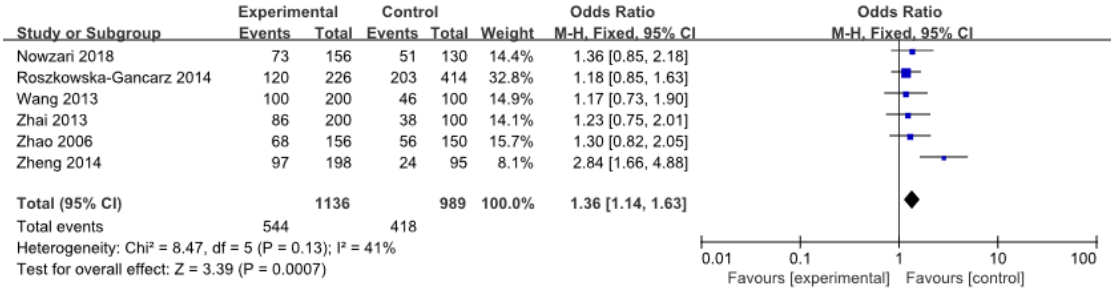

Forest plot of the ***LEP*** **rs7799039** polymorphism and CAD under additive comparison.

(GA vs. GG + AA)

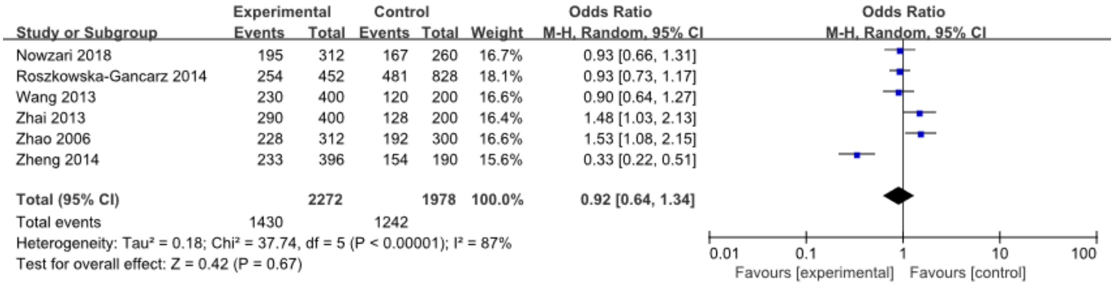

Forest plot of the ***LEP*** **rs7799039** polymorphism and CAD under allele comparison.

(G vs. A)

Supplementary figure 4-Funnel plots of LEPR rs1137100

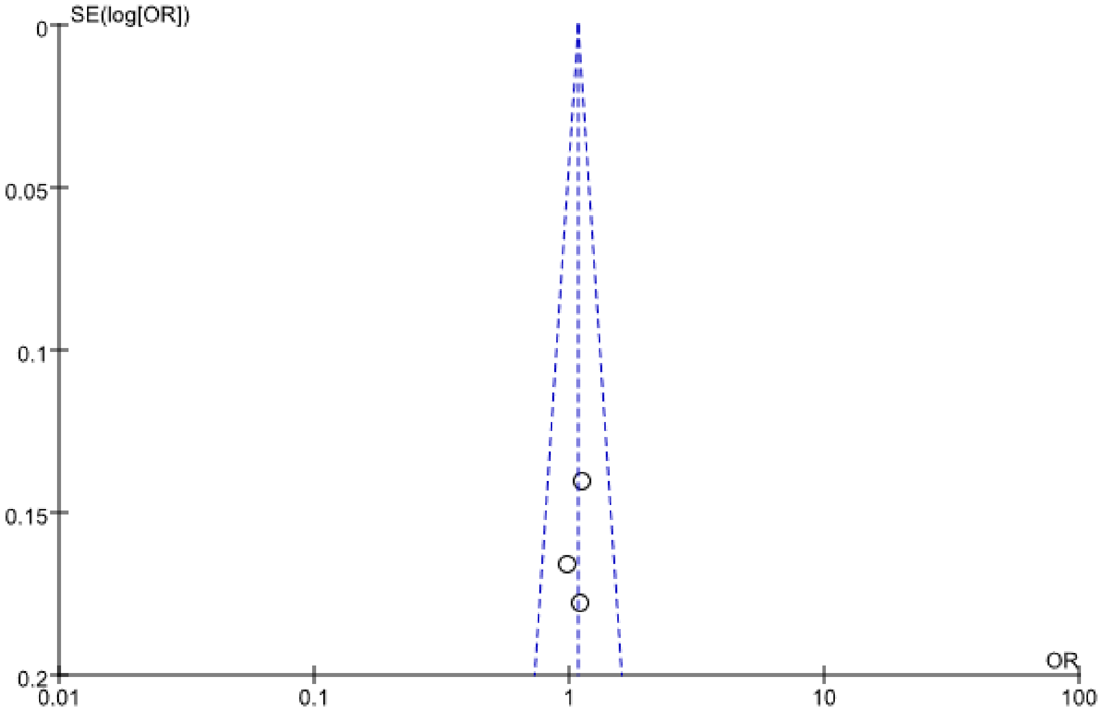

Funnel plot of the ***LEPR*** **rs1137100** polymorphism and CAD under dominant comparison.

(AA vs. AG + GG).

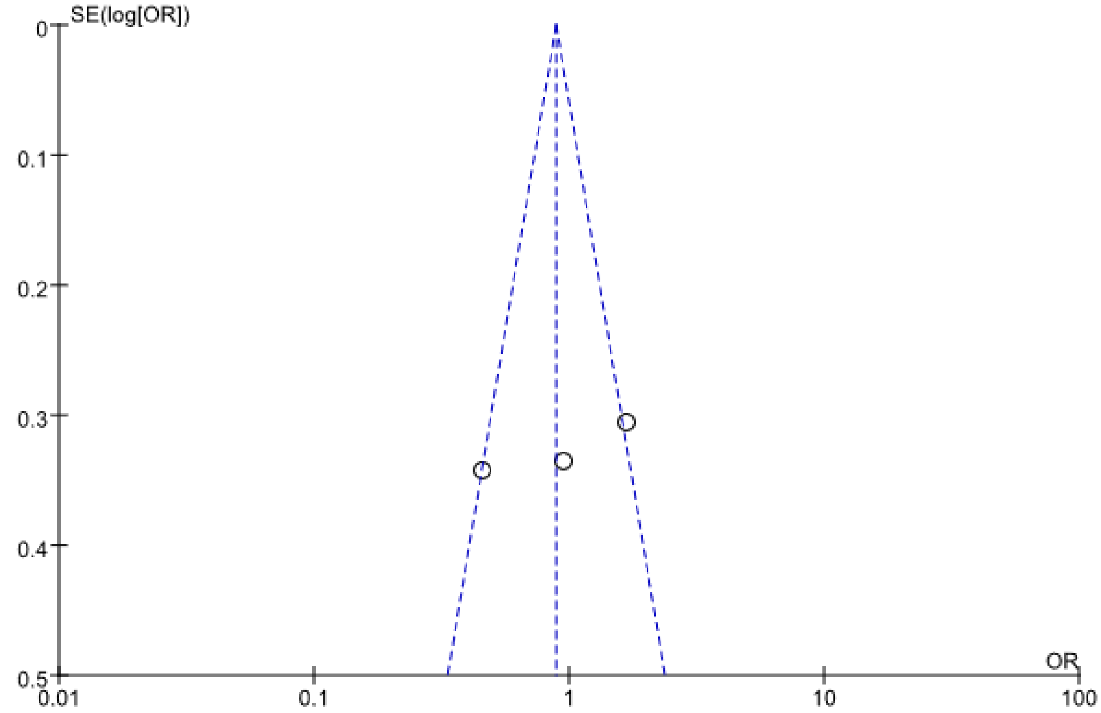

Funnel plot of the ***LEPR*** **rs1137100** polymorphism and CAD under recessive comparison.

(GG vs. AA + AG)

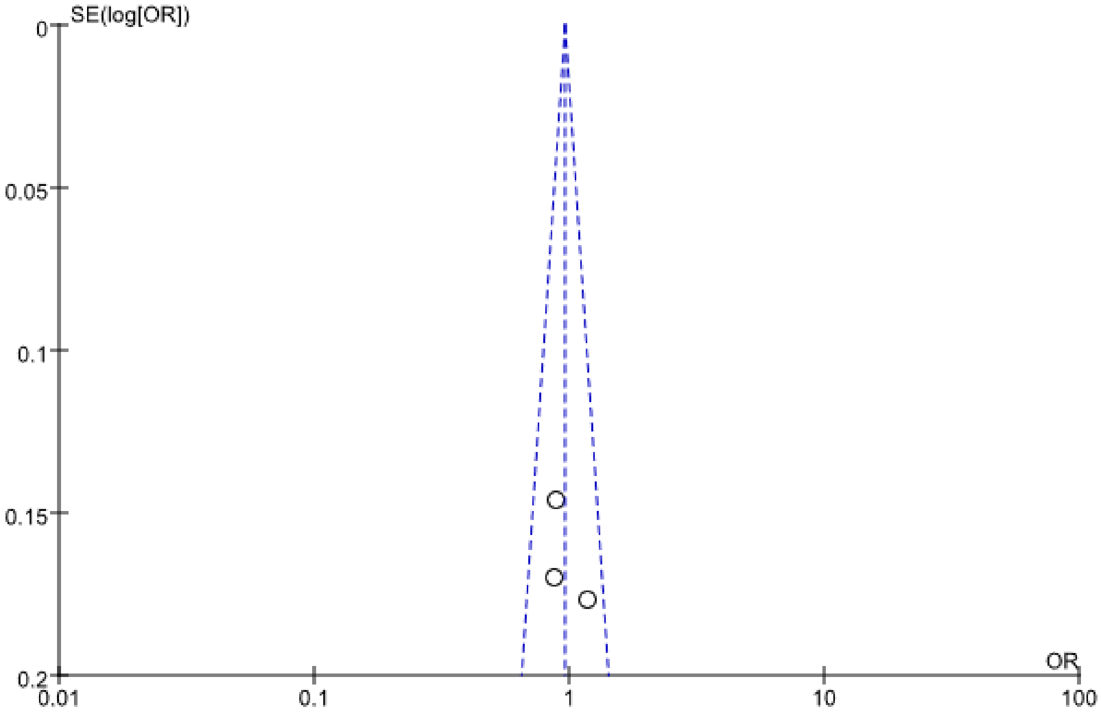

Funnel plot of the ***LEPR*** **rs1137100** polymorphism and CAD under additive comparison.

(AG vs. AA + GG)

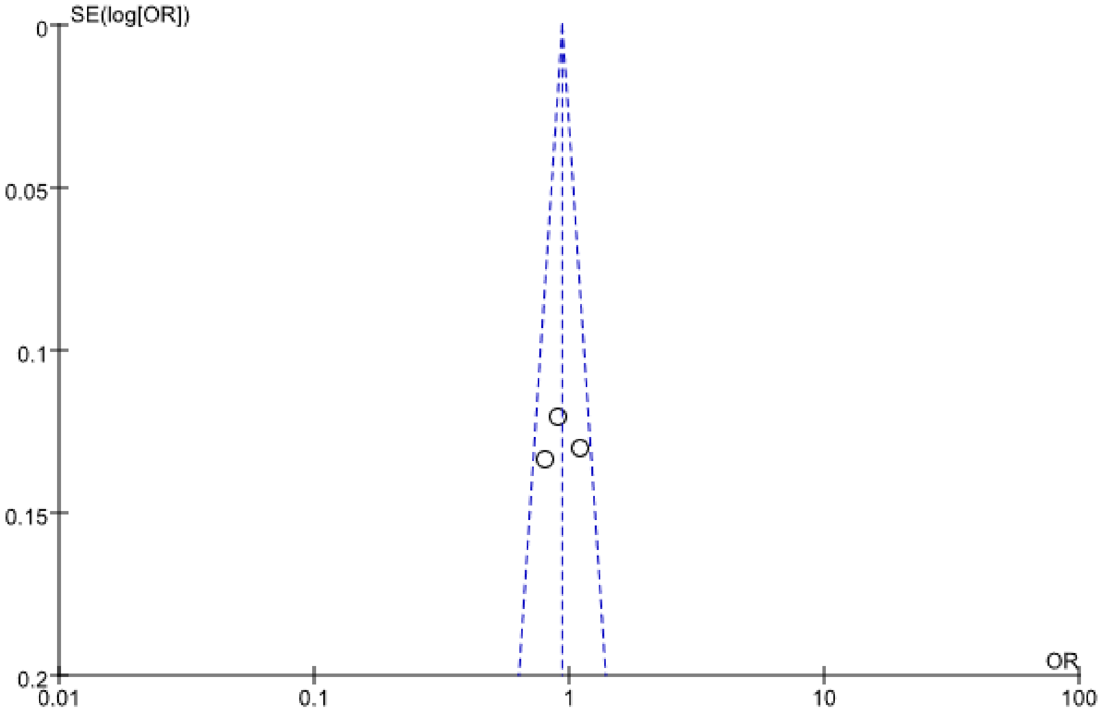

Funnel plot of the ***LEPR*** **rs1137100** polymorphism and CAD under allele comparison.

(A vs. G)

Supplementary figure 5-Funnel plots of LEPR rs1137101

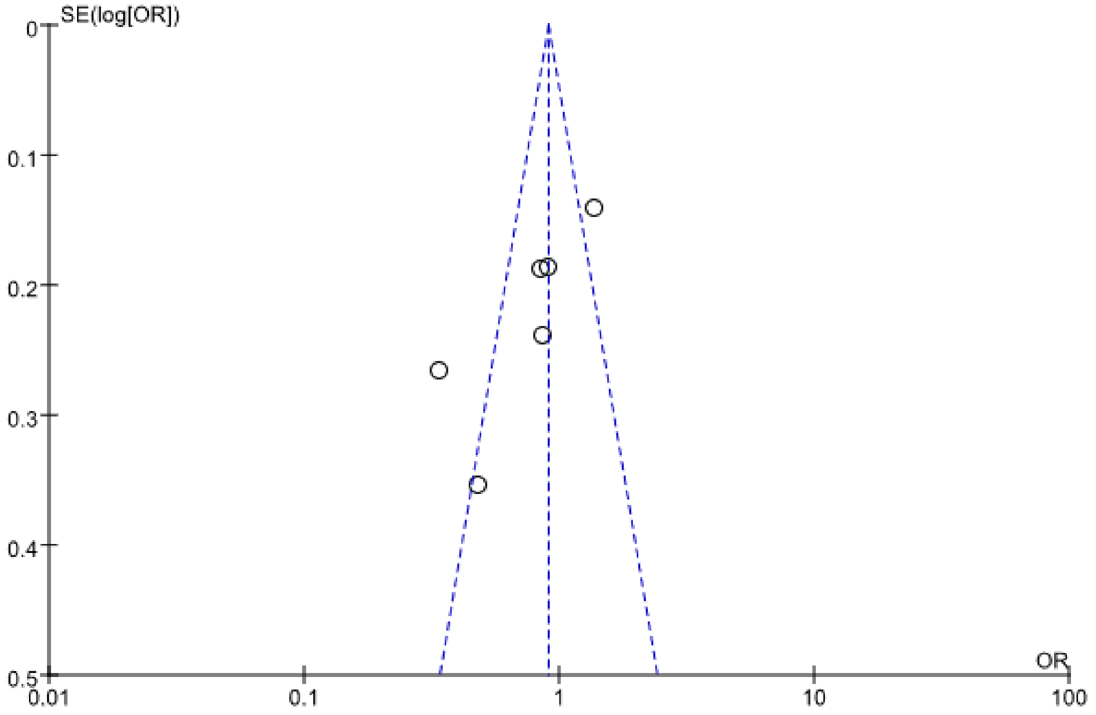

Funnel plot of the ***LEPR* rs1137101** polymorphism and CAD under dominant comparison.

(GG vs. GA + AA)

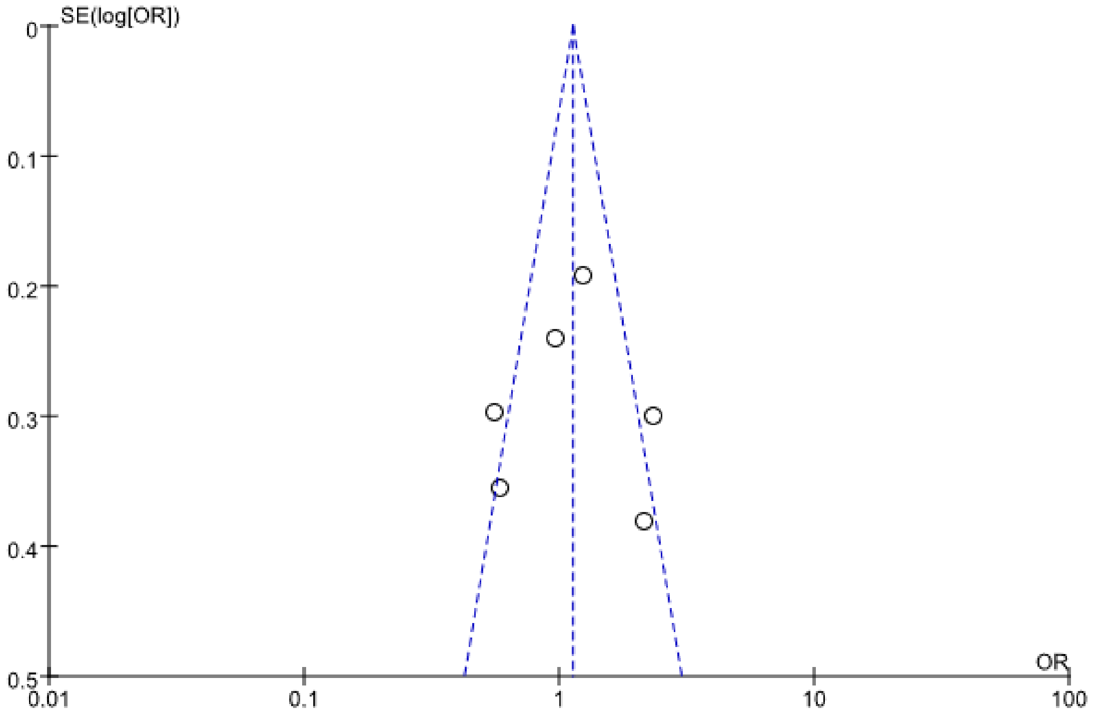

Funnel plot of the ***LEPR* rs1137101** polymorphism and CAD under recessive comparison.

(AA vs. GG + GA)

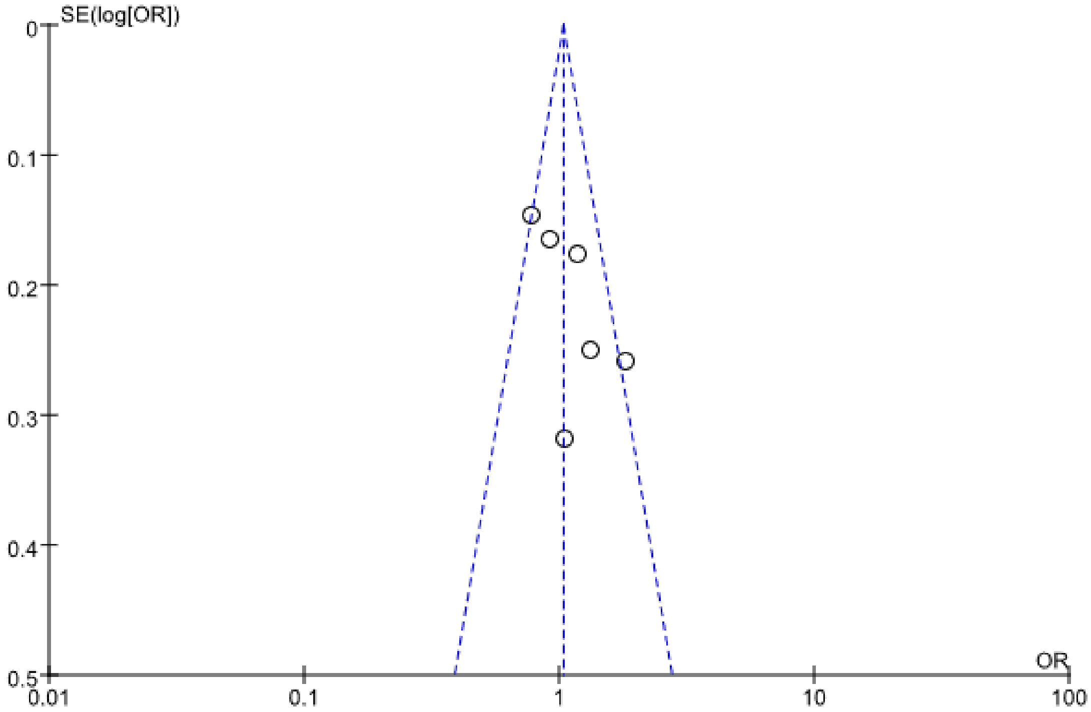

Funnel plot of the ***LEPR*** **rs1137101** polymorphism and CAD under additive comparison.

(GA vs. GG + AA)

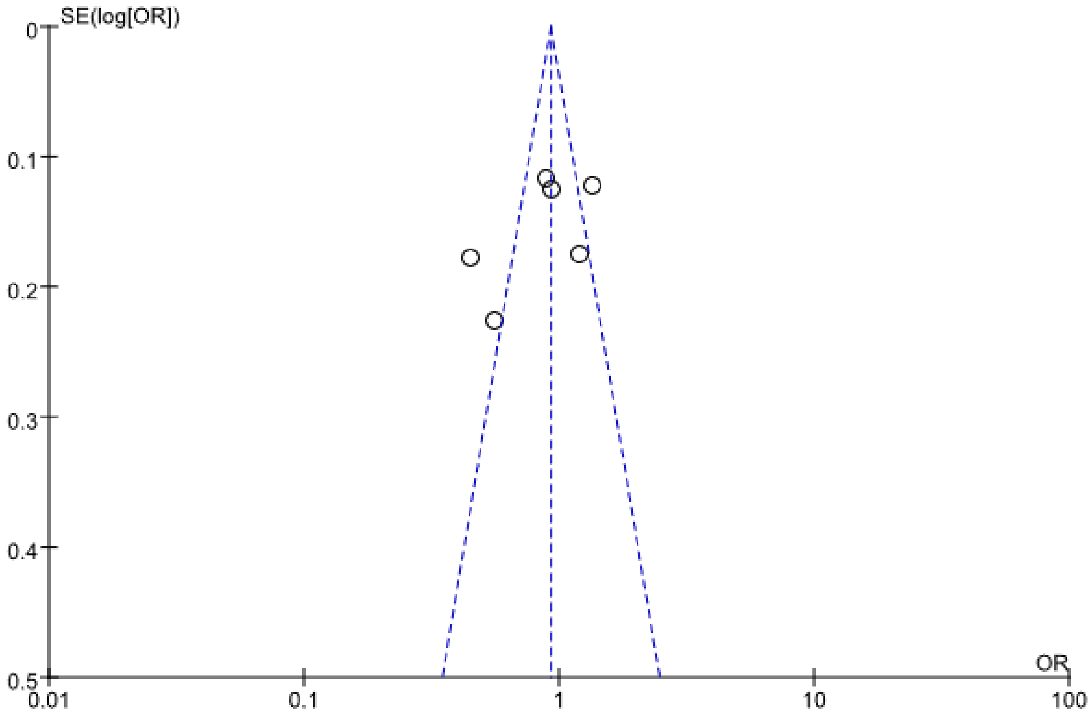

Funnel plot of the ***LEPR*** **rs1137101** polymorphism and CAD under allele comparison.

(G vs. A)

Supplementary figure 6-Funnel plots of LEP rs7799039

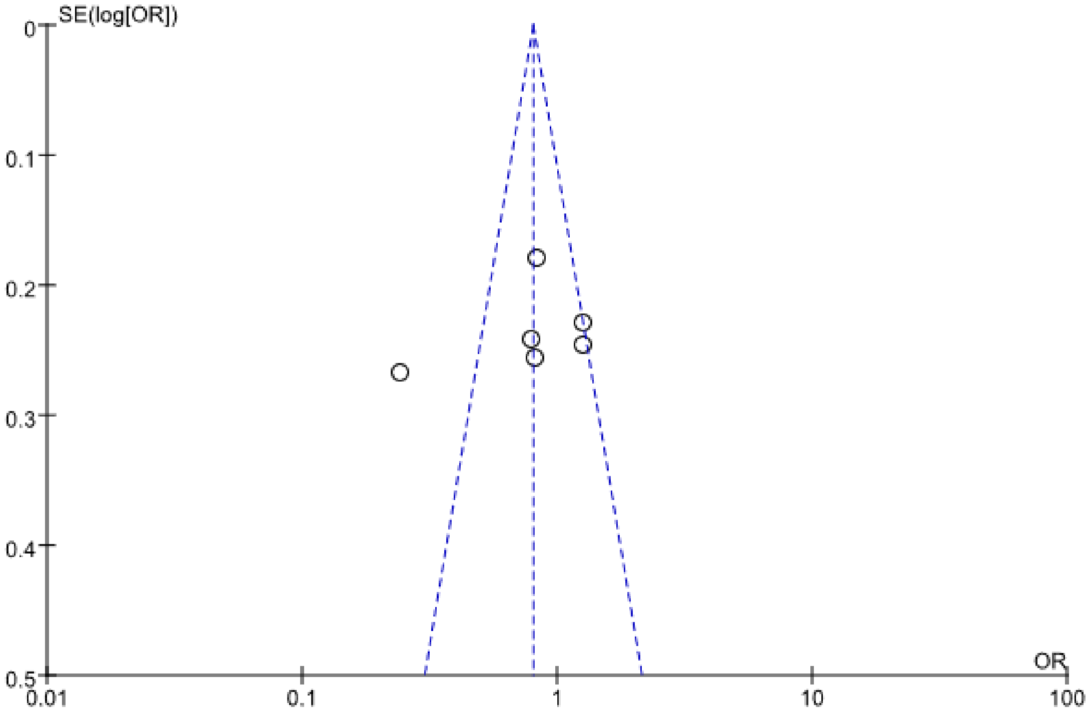

Funnel plot of the ***LEP*** **rs7799039** polymorphism and CAD under dominant comparison.

(GG vs. GA + AA)

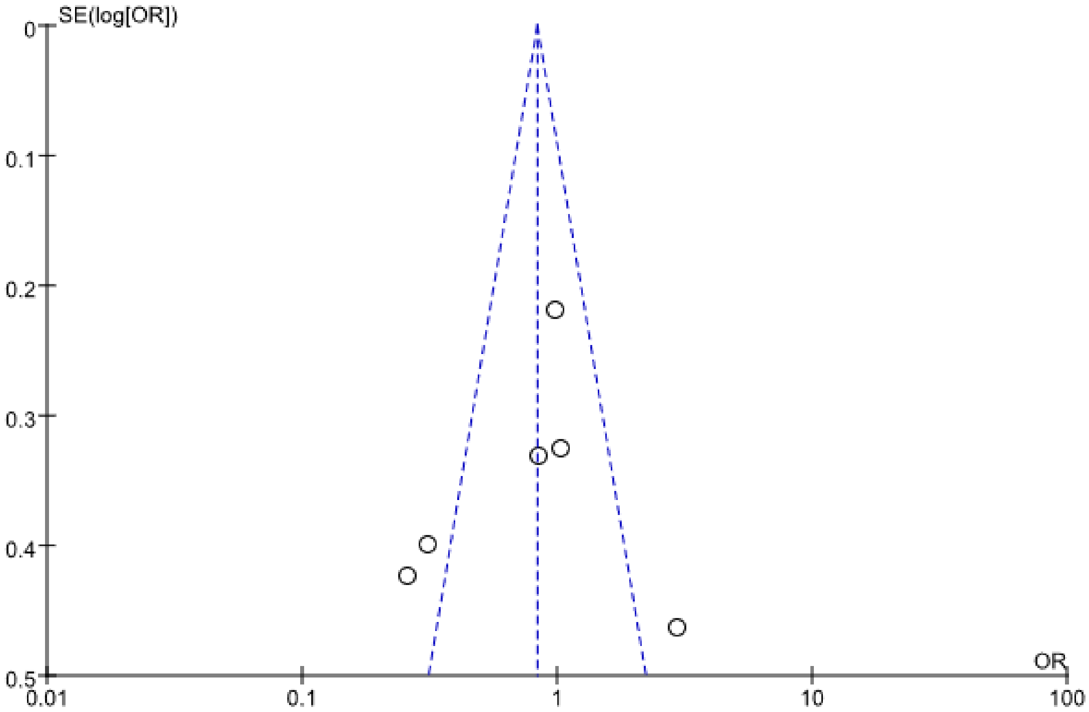

Funnel plot of the ***LEP*** **rs7799039** polymorphism and CAD under recessive comparison.

(AA vs. GG + GA)

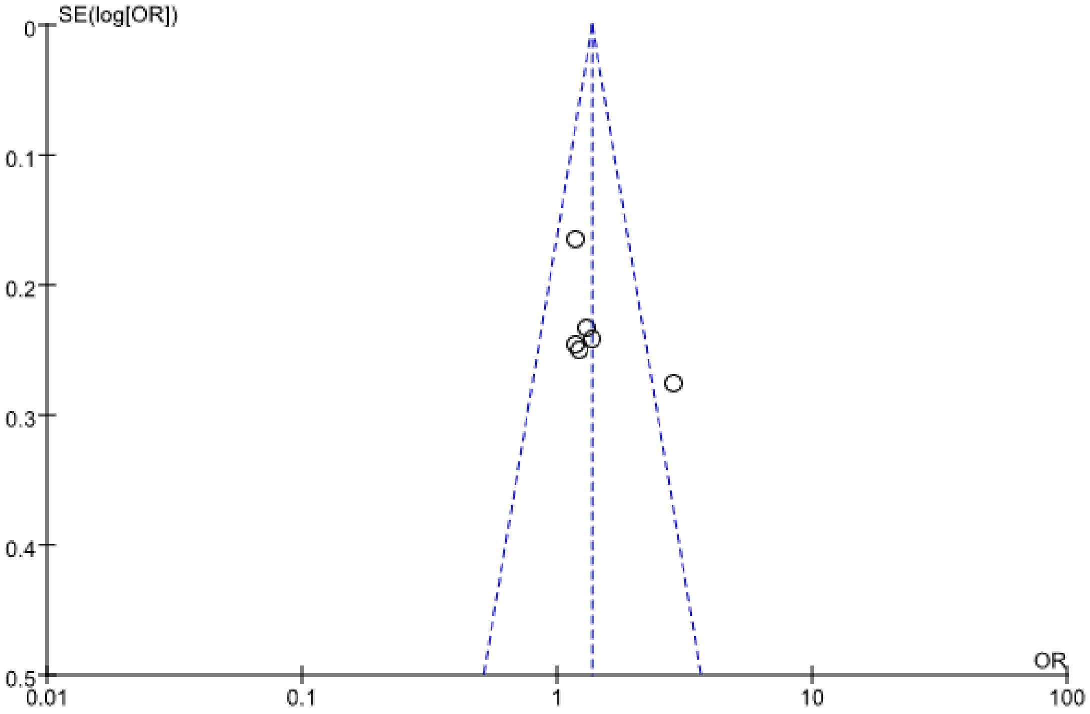

Funnel plot of the ***LEP*** **rs7799039** polymorphism and CAD under additive comparison.

(GA vs. GG + AA)

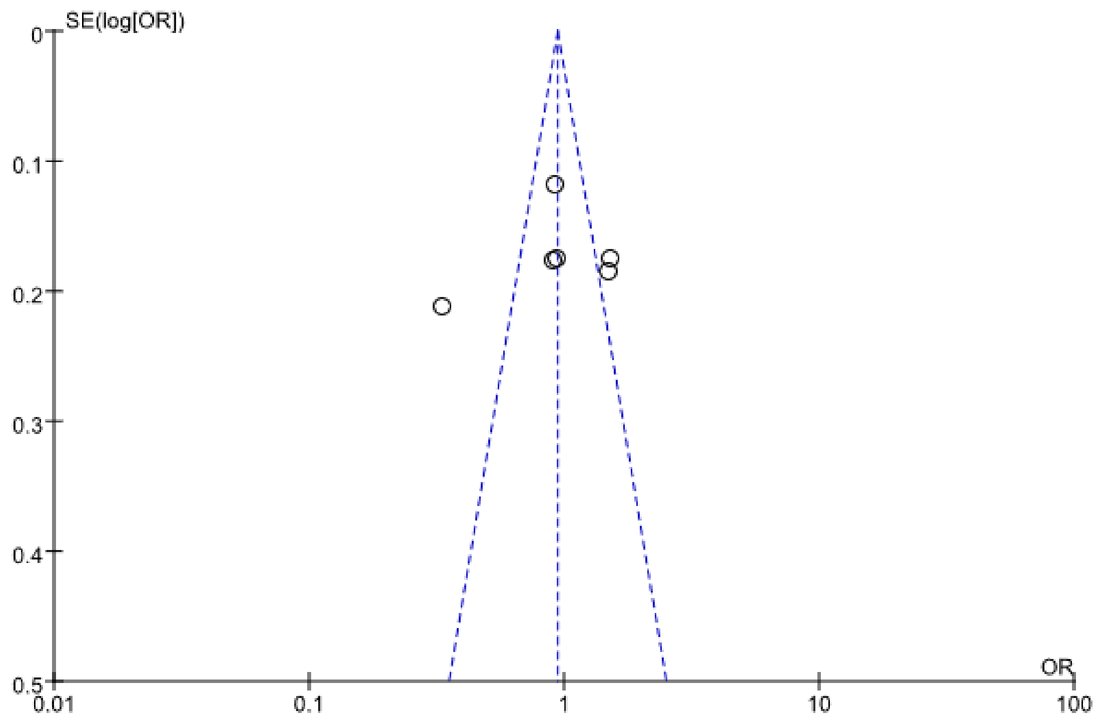

Funnel plot of the ***LEP*** **rs7799039** polymorphism and CAD under allele comparison.

(G vs. A)
